# Transient early neurotrophin release and delayed inflammatory cytokine release by microglia in response to PAR-2 stimulation

**DOI:** 10.1186/1742-2094-9-142

**Published:** 2012-06-25

**Authors:** Chen-wen Chen, Qian-bo Chen, Qing Ouyang, Ji-hu Sun, Fang-ting Liu, Dian-wen Song, Hong-bin Yuan

**Affiliations:** 1 Department of Anesthesiology, Changzheng Hospital, Second Military Medical University, Shanghai, 200003, China; 2 Department of Anesthesiology, Eastern Hepatobiliary Surgery Hospital, Second Military Medical University, Shanghai, 200433, China; 3 Department of Physiology, College of Basic Medical Sciences, Second Military Medical University, Shanghai, 200433, China; 4 Department of Orthopedics, Changzheng Hospital, Second Military Medical University, Shanghai, 200003, China

**Keywords:** PAR-2, Microglia, BDNF, NO, Neuronal cell death

## Abstract

Activated microglia exerts both beneficial and deleterious effects on neurons, but the signaling mechanism controlling these distinct responses remain unclear. We demonstrated that treatment of microglial cultures with the PAR-2 agonist, 2-Furoyl-LIGRLO-NH2, evoked early transient release of BDNF, while sustained PAR-2 stimulation evoked the delayed release of inflammatory cytokines (IL-1β and TNF-α) and nitric oxide. Culture medium harvested during the early phase (at 1 h) of microglial activation induced by 2-Furoyl-LIGRLO-NH2 (microglial conditioned medium, MCM) had no deleterious effects on cultured neurons, while MCM harvested during the late phase (at 72 h) promoted DNA fragmentation and apoptosis as indicated by TUNEL and annexin/PI staining. Blockade of PAR-1 during the early phase of PAR-2 stimulation enhanced BDNF release (by 11%, small but significant) while a PAR-1 agonist added during the late phase (24 h after 2-Furoyl-LIGRLO-NH2 addition) suppressed the release of cytokines and NO. The neuroprotective and neurotoxic effects of activated microglial exhibit distinct temporal profiles that are regulated by PAR-1 and PAR-2 stimulation. It may be possible to facilitate neuronal recovery and repair by appropriately timed stimulation and inhibition of microglial PAR-1 and PAR-2 receptors.

## Background

Microglias are the major resident immunocompetent cells in the central nervous system (CNS). Microglia activation in response to trauma, metabolic insult, infection, or neurodegenerative processes can both enhance neuroprotection and contribute to neuropathogology [1]. Indeed, activated microglia are part of an endogenous system promoting restoration of endangered CNS elements [2,3], while excessive and sustained stimulation of these cells contributes to neuropathology by causing the release of inflammatory mediators and neurotoxins [4-6]. The development of treatment strategies aimed at optimizing the protective actions and minimizing the neurotoxic effects of activated microglia remains a substantial clinical challenge.

Protease-activated receptors (PARs) are a unique family of four G protein-coupled receptors (PAR-1, -2, -3, and −4) [7]; PAR-1, PAR-3, and PAR-4 are activated by thrombin, whereas PAR-2 is activated by trypsin and tryptase [7]. All members are widely expressed in the CNS by neurons, microglia, astrocytes, and oligodendrocytes, and are possible therapeutic targets for the treatment of neurodegenerative disorders [8-12]. Under pathological conditions, PARs have been shown to mediate either neuronal death or survival. Stimulation of neuronal PAR-2 protected human neurons against Aβ in vitro [13]. In contrast, activation of microglial PAR-2 receptors promoted the secretion of neurotoxic factors like nitrite, reactive oxygen species (ROS), and TNF-α, and the conditioned media from microglia induced cell death in rat primary neuronal cultures [13,14]. It is known that activation of microglial PAR-2 promotes the expression of the ATP-gated ion channel P2X4R, which in turn can trigger the release of neuroprotective brain-derived neurotrophic factor (BDNF) [15-17]. Neurotrophic factors like BDNF mediate axon guidance during neural development and can enhance axonal growth following injury [18].

Whether activation of PAR-2 in microglia is detrimental or beneficial to neurons remains equivocal. Watanabe, *et al*. found that microglia were neuroprotective only during the early phase of activation and that this effect disappeared by the late phase [19]. The mechanisms for this phenotypic transition are not known, but circumstantial evidence implicates PAR-1 and PAR-2 receptors. There is solid evidence that PAR-1 and PAR-2 act as co-factors and heterodimerization partners during signal transduction. Kaneider and coworkers demonstrated that PAR-1 switches from being a vascular disruptive to a vascular protective receptor during the progression of sepsis in mice and that this functional transition was dependent on PAR-2 [20]. Clinical findings also demonstrated that patients with diarrhea-predominant irritable bowel syndrome exhibited changes in PAR-1/PAR-2 expression ratio in the colon [21].

We hypothesized that the balance between PAR-1 and PAR-2 activation in microglia may mediate the functional transition from a neuroprotective to neurotoxic phenotype. To clarify the roles of PAR-1 and PAR-2 on microglial function, we examined the time-dependent changes in neurotrophic and inflammatory responses following activation by the PAR-2 agonist.

## Materials and methods

### Animals

Male and female Sprague Dawley (SD) rat pups from 1 to 3 day-old and E16-17 fetal SD rat embryos were obtained from the SLAC laboratory animal center, Shanghai, China. The treatment of all rats used in this study conformed to the Guide for the Care and Use of Laboratory Animals (National Institutes of Health publication number 80–23) as approved by the Ethics Committee for Animals of the Second Military Medical University.

### Cell cultures and experimental treatments

Microglia was isolated and cultured from 1 to 3 day-old SD rats as previously described [22]. Briefly, the cerebral cortices were triturated into a single cell suspension in DMEM-Ham‘s F-12 medium (GIBCO-BRL, Grand Island, NY, USA) supplemented with 10% fetal bovine serum (GIBCO-BRL, Grand Island, NY, USA ), 2 mM L-glutamate, and 1 mM sodium pyruvate. Cells were plated in 75 cm^2^ T-flasks and incubated for 14 days. Microglia were detached from the flasks by mild shaking, and filtered through a nylon mesh to remove astrocytes and clumped cells. Purified microglia were plated on 60 or 100 mm dishes at 0.1-5.0 × 10^6^ cells/dish.

Cultured microglia were incubated with 10 μM of the PAR-2 agonist 2-Furoyl-LIGRLO-NH2 (Sigma-Aldrich, St. Louis, MO, USA) to induce activation. Microglia conditioned medium (MCM) was collected after 0.0, 0.5, 1.0, 2.0, 12.0, 24, or 72.0 h of continuous 2-Furoyl-LIGRLO-NH2 stimulation. The levels of nitrite, BDNF, IL-1β, and TNF-α in the MCM were determined by the assays described in the next subsection. We also examined the effects of PAR-1 activation and blockade on microglial neurotrophin release during the early phase of activation and cytokine release during the late phase of activation. To examine the effect of PAR-1 activity during the early phase of activation, microglia were stimulated by 2-Furoyl-LIGRLO-NH2, 2-Furoyl-LIGRLO-NH2 plus the PAR-1 antagonist SCH79797 (Axon Medchem, Groningen, NL), or 2-Furoyl-LIGRLO-NH2 plus the PAR-1 agonist TFLLR (Tocris Bioscience, Anonmouth, UK). The three MCM samples were collected after 1 h of stimulation and the BDNF concentrations assayed as described in the next subsection. To examine the effect of PAR-1 activity on microglial secretion of cytokines and nitric oxide (NO) during the late phase, microglia were stimulated with 2-Furoyl-LIGRLO-NH2 for 24 h, followed by addition of SCH79797 or TRLLR. The MCM was collected 48 h after agonist or antagonist application (or 72 h after the onset of 2-Furoyl-LIGRLO-NH2 stimulation) and assayed as described below.

Primary rat neurons were prepared from E16 SD rat embryos as previously described [23]. Briefly, the cerebral cortex was dissected and transferred into Ca^2+^- and Mg^2+^-free D-Hank's solution. The dissected tissues were incubated in trypsin solution at 37°C and dissociated by trituration. The dissociated cells were re-suspended in B27-supplemented neurobasal medium (Invitrogen, Carlsbad, CA, USA) and plated onto 60 mm culture dishes. Cultured cortical neurons were incubated for 10 days at 37°C under 10% CO_2_ before experiments.

To examine the effects of MCM collected at different times after the start of PAR-2 stimulation on neuronal viability, cultured neurons were incubated for 48 h in MCM from unstimulated microglia (control condition), MCM collected after 1 h of 2-Furoyl-LIGRLO-NH2 stimulation (1 h MCM or early phase MCM), or MCM collected after 72 h of 2-Furoyl-LIGRLO-NH2 stimulation (72 h MCM or late phase MCM). The neurotoxicity of these supernatants was estimated by transferase-mediated dUTP nick-end labeling (TUNEL) assay and flow cytometry as described below.

### Transfection and clone selection

The PAR-2 shRNA (r) lentiviral particles were purchased from Santa Cruz Biotechnology, Inc. Control shRNA lentiviral particles were used as controls. The lentiviral particles were transfected into cells according to the manufacturer's instructions. Stably transfectant clones were selected via Puromycin dihydrochloride (Sigma-Aldrich, St. Louis, MO, USA) selection and were validated by quantitative real-time polymerase chain reaction (qRT-PCR) and immunoblotting.

### Realtime PCR

An RNeasy Mini Kit (Qiagen, Shanghai, CHN) was used to extract RNA from cells. Total RNA (200 ng) and oligo-dT primers were used to synthesize single-stranded cDNA using Qiagen’s Omniscript reverse transcriptase according to the manufacturer’s instructions. The resulting cDNA was purified and PCR was set up with an ABI Prism 7900 Sequence Detection System and SYBR green Master Mix (Applied Biosystems, Carlsbad, CA, USA). The primers used were as follows: 5’- CGTGCTGCTCGTCGTGCATTATTT-3’ and 5’- TTTCTGGCCTGGTCCCTGAAATCT-3’ for PAR2, 5’- TTGCTGACAGGATGCAGAAGGAGA -3’ and 5’- ACTCCTGCTTGCTGATCCACATCT -3’ for β-actin. The relative expression of PAR2 mRNA was calculated with the 2 to the power of minus Delta Delta CT (2_T_^-△△C^) method, using β-actin mRNA expression level for normalization.

### Western blotting

Cells were homogenized and scraped in lysis buffer (50 mM Tris_HCl, pH 8.0, 1 mM EDTA, 1% Triton X-100, 0.5% sodium deoxycholate, 0.1% SDS, 150 mM NaCl, 29 protease inhibitor mix). Then the samples were centrifuged and the supernatant was collected. The proteins were separated by sodium dodecyl sulfate polyacrylamide gel electrophoresis (SDS-PAGE and transferred to polyvinylidene difluoride (PVDF) membrane, and the specific proteins were detected by immunoblotting. Primary antibodies against PAR-2 (sc-13504, Santa Cruz, CA, USA) and β-actin (sc-81178, Santa Cruz, CA, USA) were used to probe the blots.

### Measurement of nitrite, BDNF, IL-1β, and TNF-α

The levels of BDNF, IL-1β, and TNF-α in culture supernatant were determined by enzyme-linked immunosorbent assay (ELISA) with specific reagent kits (R&D Systems, Inc., USA) following the manufacturer’s instructions. Nitric oxide production was calculated from the amount of nitrite detected in the media by the Griess reaction as reported previously [24]. For statistical analysis, data were obtained from at least three individual experiments.

### Cell viability assay

Cell viability of cultured microglial cells were measured by quantitative colorimetric assay with 3-(4,5)-dimethylthiahiazo(−z-y1)-3,5-di-phenytetrazoliumromide (MTT) (Sigma-Aldrich, St. Louis, MO, USA). The MTT assay relies primarily the mitochondrial activity of living cells and reflects the intracellular redox state. Upon termination of the experiments, the culture media were aspirated and MTT (final concentration, 1 mg/ml) was added to cells and then incubated at 37°C for 2 h. The supernatant was aspirated and dimethyl sulfoxide (Sigma-Aldrich, St. Louis, MO, USA) was added to the wells. Insoluble crystals were dissolved by mixing and the plates were read on an absorbance reader, using a test wavelength of 570 nm and a reference wavelength of 630 nm. Results were expressed as the percentage (%) of MTT reduction, assuming that the absorbance of control cells was 100%.

### Terminal deoxynucleotidyl transferase-mediated dUTP nick-end labeling (TUNEL)

Fragmentation of DNA in early apoptotic neurons was assayed using a commercially available *in situ* death detection kit (product of Roche Diagnostic). Neurons (1 × 10^5^ cells/ml) were treated as described and fixed for 30 min in 10% neutral buffered-formalin solution at room temperature. For TUNEL labeling, the *in situ* cell death detection kit (fluorescein) from Roche Applied Science was used according to the manufacturer’s instructions. Staining with 4,6-diamidino-2-phenylindole (DAPI) was performed using a ready-to-use mounting medium containing DAPI. Labeled cells were rinsed with phosphate-buffered saline (PBS) and examined under a confocal microscope with excitation at 488 nm and emission at 525 nm. Percentage of apoptotic cells was calculated by counting in at least 6 randomly chosen subfields. Results are mean ± standard error (SE) of counting in three independent experiments.

### Measurement of apoptotic cell death by flow cytometry

Rat cultured neurons (5 × 10^5^/dish) were treated with 6.84 mM EDTA (Sigma-Aldrich, St. Louis, MO, USA) and 0.05% trypsin (Sigma-Aldrich, St. Louis, MO, USA) for 5 to 7 minutes. The cells were detached by shaking in order to bring them into suspension. The cells were then harvested by centrifuging at 200 × g for 5 minutes. The cell pellet was re-suspended in 100 μl of 1 mg/ml annexin-V-FITC in HEPES (N-2-hydroxyethylpiperazine-N'-2-ethanesulphonic acid) buffer with 1.8 mM CaCl_2_. The suspension was incubated for 5 to 10 minutes at room temperature in the dark, followed by addition of 1 ml HEPES containing 10 mg/ml propidium iodide (PI). The fraction of apoptotic cells was analyzed immediately by flow cytometry (Becton Dickinson, San Jose, CA).

### Statistical analysis

Mean values were calculated from three or more independent experiments and are reported as mean ± SE. Group means were compared by one-way analysis of variance (ANOVA), followed by post hoc pair-wise comparisons using Student-Newman-Keuls tests. A *P* value < 0.05 was defined as statistically significant.

## Results

### The PAR-2 agonist 2-furoyl-LIGRLO-NH2 induced early release of BDNF and delayed release of proinflammatory cytokines from rat microglia

**Figure 1:**
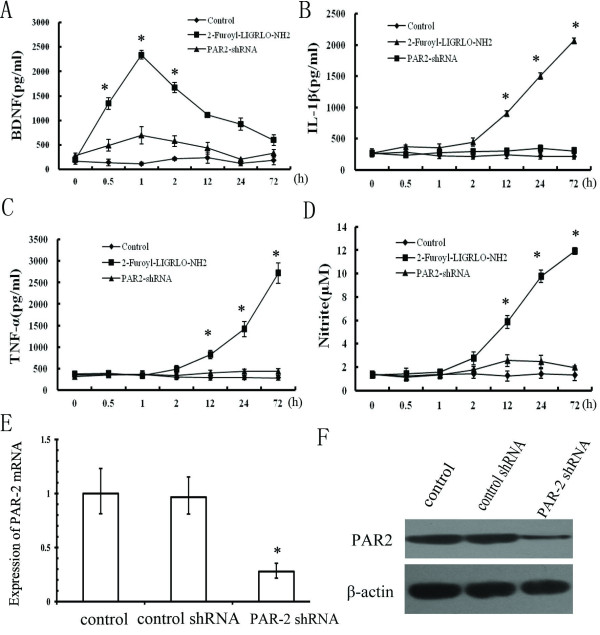
**Release of neurotrophic and proinflammatory factors from PAR-2 activated rat microglia.** Cells were treated with 10 μM 2-Furoyl-LIGRLO-NH2 in the presence or absence of PAR-2 shRNA. The concentration of BDNF, IL-1β, TNF-α, and NO in the supernatant were determined at 0.0, 0.5, 1.0, 2.0, 12.0, 24.0, and 72.0 h after 2-Furoyl-LIGRLO-NH2 application as described previously (**A**, **B**, **C**, **D**). All values represent means ± SE. *Significantly different from control group (*P* < 0.05). qRT-PCR and immunoblot analysis were also shown to verify the decreased expression of PAR-2 in the PAR-2 shRNA treated rat microglia (**E**, **F**).

To examine the distinct neurotrophic and inflammatory responses of microglia after PAR-2 stimulation, cultured microglia were incubated with the PAR-2 agonist 2-Furoyl-LIGRLO-NH2 (Figure 1). Microglia conditioned medium (MCM) was collected after 0.0, 0.5, 1.0, 2.0, 12.0, 24.0, and 72.0 h of 2-Furoyl-LIGRLO-NH2 stimulation and the amount of BDNF, IL-1β, and TNF-α released was measured by ELISA. The concentration of BDNF rose significantly during the first 1 h of 2-Furoyl-LIGRLO-NH2 treatment, but returned to near baseline within 72 h. In contrast, the concentrations of the proinflammatory factors IL-1β and TNF-α did not increase until 12 to 24 h after the start of 2-Furoyl-LIGRLO-NH2 treatment and remained elevated for at least 72 h. Similarly, nitrate concentration, a proxy for NO release, also rose after about 12 h of 2-Furoyl-LIGRLO-NH2 treatment and continued to increase for the duration of the measurement period. Thus, neuroprotective BDNF was released from microglia during the early phase of the activation response while proinflammatory cytokines and cytotoxic NO were only released in response to sustained PAR-2 activation.

When the PAR-2 shRNA expression vector was transfected into the microglia, the expression of PAR-2 was reduced significantly. In this condition, increased release of BDNF, cytokine, and NO induced by 2-Furoyl-LIGRLO-NH2 was inhibited significantly, indicating that all these secretory responses were mediated by PAR-2 activation.

### The MCM from late-phase PAR-2-activated microglial was neurotoxic

**Figure 2:**
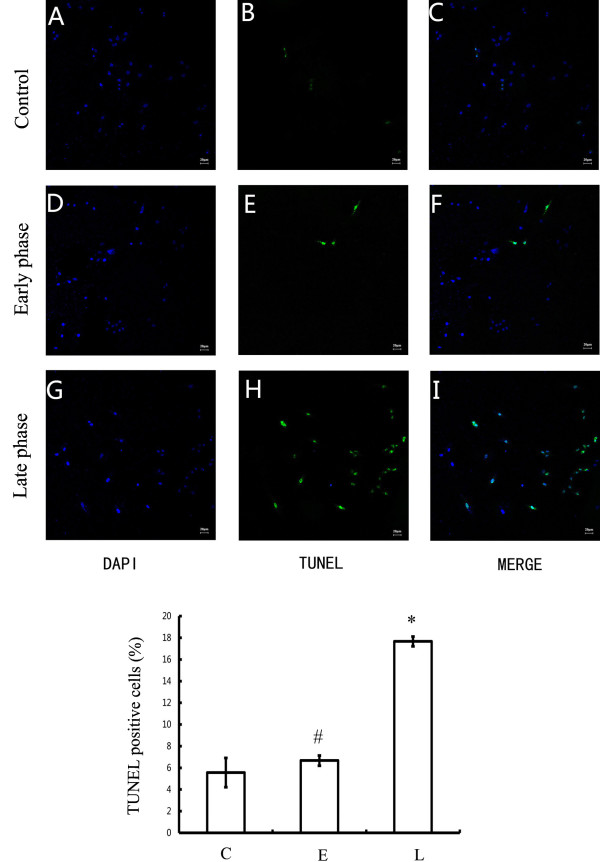
**Stage-specific effect of MCM on nuclear DNA fragmentation in rat cortical neurons.** Neurons were incubated in unstimulated MCM (control), MCM at 1 h after 10 μM 2-Furoyl-LIGRLO-NH2 stimulation (early phase) and MCM at 72 h after stimulation (late phase) for 48 h. Internucleosomal DNA fragmentation was determined by a fluorescent TUNEL assay. Percentage of apoptotic cells was calculated by counting in at least 6 randomly chosen subfields. Values are means ± SE. *Significantly different from control group, *P* < 0.05; # No significantly different from control group, *P* > 0.05.

To evaluate the effects of these microglial factors on neuronal viability, the media from 2-Furoyl-LIGRLO-NH2-treated microglia (MCM) was retrieved and used to treat cultured cortical neurons. Cultured neurons were incubated for 48 h in MCM collected from microglial cultures after 0, 1, or 72 h of 2-Furoyl-LIGRLO-NH2 stimulation. Neuronal viability was assessed by the TUNEL assay for DNA fragmentation associated with activation of apoptotic signaling cascades (Figure 2). The number of TUNEL-positive cells was significantly higher in neuronal cultures incubated in 72 h MCM than in cultures incubated in media from unstimulated microglia (control MCM), while the number of TUNEL-positive neurons in cultures incubated with 1 h MCM was not significantly different from cultures incubated in control MCM. Consistent with the results presented in Figure 1, the MCM from 2-Furoyl-LIGRLO-NH2-stimulated microglia only induced apoptosis when microglia entered the late phase of the reactive response associated with the release of inflammatory cytokines and NO.

**Figure 3:**
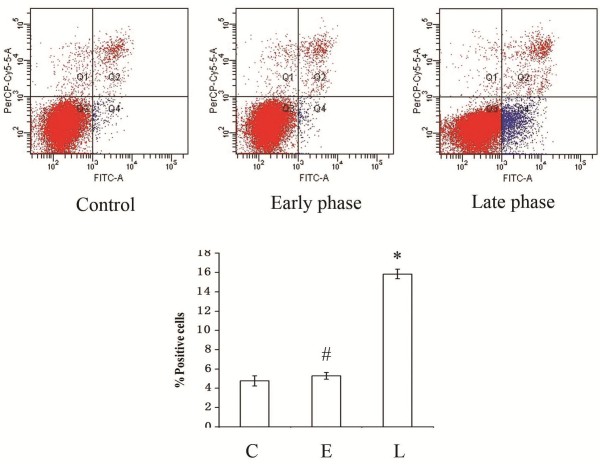
**Stage-specific effect of MCM on apoptotic cell death in rat cortical neurons.** Neurons were exposed to unstimulated MCM (control), MCM at 1 h after 10 μM 2-Furoyl-LIGRLO-NH2 stimulation (early phase) and MCM at 72 h after stimulation (late phase) for 48 h. The percentage of early apoptotic and late apoptotic cells was determined by fluorescence activated cell sorting (FACS). Data are means ± SE. *Significant differences from control group, *P* < 0.05; # No significant difference from control group: *P* > 0.05.

An annexin V-FITC/PI apoptosis detection kit was used to quantify the percentage of apoptotic neurons following MCM treatment. Three populations of neurons were distinguished by this dual staining method: viable (no staining), early apoptotic (Annexin V ^+^ PI^−^), and late apoptotic (Annexin V ^+^ PI^+^). Flow cytometry demonstrated that the percentage of early apoptotic and late apoptotic neurons incubated in 72 h MCM was significantly higher than in cultures treated with 1 h MCM or MCM from untreated microglial cultures (Figure 3).

### Blockade and activation of PAR-1 altered PAR-2-induced BDNF and cytokine release from microglia

**Figure 4:**
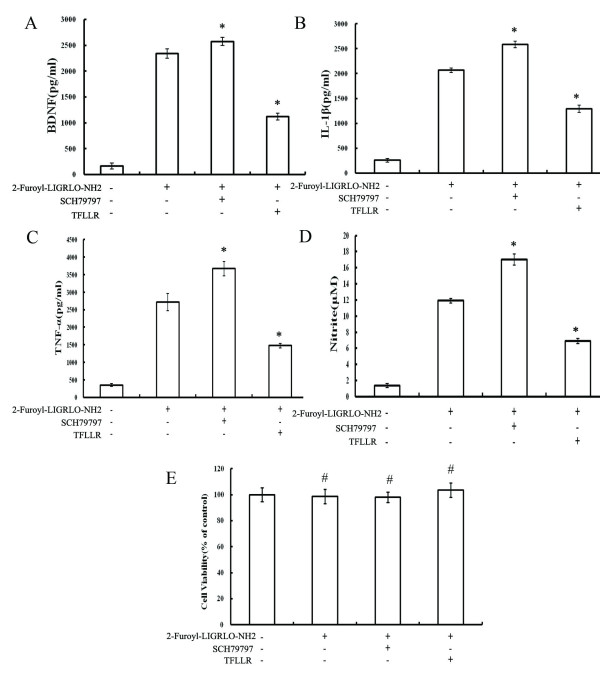
**Stage-specific effect of a PAR-1 agonist and antagonist on neurotrophic and proinflammatory responses in rat microglia activated by 2-Furoyl-LIGRLO-NH2.** (**A**) Microglia was stimulated by 2-Furoyl-LIGRLO-NH2 (10 μM), 2-Furoyl-LIGRLO-NH2 plus SCH79797 (1 μM), or 2-Furoyl-LIGRLO-NH2 (10 μM) plus TFLLR (0.5 μM). The concentration of BDNF in MCM that was collected at 1 h after stimulation was determined by ELISA. Values are means ± SE. *Significantly different from 2-Furoyl-LIGRLO-NH2 treated group, *P* < 0.05. (**B**, **C**, **D**) Microglia was stimulated by 2-Furoyl-LIGRLO-NH2, then SCH79797 or TRLLR was added 24 h later (during the late phase of microglial activation). The concentration of IL-1β, TNF-α, and NO in MCM collected at 72 h after 2-Furoyl-LIGRLO-NH2 stimulation were determined as described. Values are means ± SE. *Significantly different from 2-Furoyl-LIGRLO-NH2 treated group: *P* < 0.05. (**E**) Cell viability was assessed by MTT assay, and the percentage of surviving cells compared to control cells. Values are means ± SE. # No significantly different from control group, *P* > 0.05.

The relative balance of PAR-1 and PAR-2 receptor expression (or stimulation) appears to mediate the phenotypic switching of resident immune cells in other tissues. To test the hypothesis that PAR-1 and PAR-2 can also regulate the distinct neuroprotective and neurotoxic temporal phases of microglial activation, we tested the effects of a PAR-1 agonist (TFLLR) and antagonists (SCH79797). MCM was collected at 0, 1, and 72 h and analyzed for BDNF, IL-1β, TNF-α, and NO metabolite content. Early blockade of PAR-1 increased the release of BDNF from microglia. In contrast, PAR-1 activation at 24 h after 2-Furoyl-LIGRLO-NH2 stimulation significantly decreased the late phase release of NO, IL-1β, and TNF-α (Figure 4). These results indicate that PAR-1 and PAR-2 receptors control the beneficial neurotrophic response and the late phase inflammatory cytotoxic response [20].

Since it has been widely reported in the literature that PAR-1 activation can modify cell proliferation and cell death, the viability of microglial cells was evaluated using the MTT test after treatment with the PAR-2 agonist in combination with PAR-1 agonist or antagonists. PAR2 agonist (2-Furoyl-LIGRLO-NH2), PAR-1 agonist (TFLLR) and antagonists (SCH79797) did not affect cell viability. Therefore, the effects of blockade and activation of PAR-1 on 2-Furoyl-LIGRLO-NH2-induced inflammation-related responses in activated microglia were not the result of the effects on cell survival.

## Discussion

Activated microglia can mediate either neuroprotection or neurotoxicity in both culture models and *in vivo*. We have provided evidence that stimulation of PAR-2 activates the release of both neurotrophic and inflammatory factors from microglia, but with distinct temporal profiles. Furthermore, we demonstrated that PAR-1 and PAR-2 can also act as co-factors to promote the beneficial anti-inflammatory phenotype during the early phase of microglial activation [20]. These data are consistent with the formation of receptor complexes or co-factoring that occurs with many members of the PAR family [25].

PAR-2 is activated by trypsin, tryptase and some synthetic peptides, such as 2-Furoyl-LIGRLO-NH2 [26]. As trypsin and tryptase may also proteolyse other molecules in cell culture, the agonist peptide 2-Furoyl-LIGRLO-NH2, which could specifically activate PAR-2, was used in our study. It has been demonstrated that stimulation of PAR-2 also triggered activation of rat primary microglial cells [14]. Microglial activation triggers the secretion of a variety of proinflammatory and neurotoxic factors, including NO, IL-1β, IL-6, and TNF-α, which can damage surrounding cells [27-29]. Previous studies have demonstrated that PAR-2 expression in local glial cells was increased in Alzheimer’s disease and paralleled upregulation of proinflammatory cytokines and chemokines [13]. Activation of microglial cells by PAR-2 stimulation produced conditioned media that were toxic to cultured rat primary neurons [14]. The neurodegenerative effects of PAR-2 stimulation are mediated by the mitogen-activated protein kinase (MAPK) pathways, including ERK1/2, c-Jun N-terminal kinase (JNK), and p38MAPK. However, it is well known that MAPKs mediate diverse biological processes and cellular responses, so we investigated the possibility that PAR-2 could induce the release of either neurotrophic or inflammatory cytokines depending on stimulus conditions. Indeed, activation of PAR-2 expressed on rat primary microglial cells triggered the early transient release of BDNF and the delayed release of inflammatory cytokines and NO.

Brain-derived neurotrophic factor is a member of the neurotrophin family of growth factors that enhance axonal growth following injury and maintain neuronal viability under a variety of stressful conditions. Thus, PAR-2-mediated activation of microglia may exert immediate but transient neuroprotective effects following insult, whereas sustained activation would lead to a delayed late phase response associated with the release of proinflammatory cytokines that contribute to neuronal cell death. Dual annexin/PI staining and TUNEL staining demonstrated a marked increase in apoptotic neurons when treated with MCM at 72 h after 2-Furoyl-LIGRLO-NH2 stimulation. This results are consistent with previous reports showing that the neuroprotective effect of microglia appears only in the early phase after glutamate treatment (a model of excitotoxicity) but disappeared in the late phase [19].

The second question addressed in this study was whether PAR-1 and PAR-2 stimulation act together to influence activated microglia phenotype. It is known that PAR-mediated signal transduction is also modulated by interactions with other PARs [25]. PAR-3 functions as a co-factor facilitating thrombin-mediated activation of PAR-4 [30,31], and PAR-1 and PAR-2 mediate crosstalk between coagulation and fibrinolysis on tumor cells [32]. In addition, PAR-1 switched from a vascular disruptive to a vascular protective receptor during sepsis in mice, a functional transition that was dependent on PAR-2 receptors. Stimulation of PAR-1 and PAR-2 might confer a beneficial anti-inflammatory microglial phenotype only within a brief time window. Greater appreciation of these distinct temporal phases of activation may allow PARs to be exploited clinically. Indeed, selective activation of PAR-1/PAR-2 complexes was beneficial for the treatment of sepsis at a specific stage [20], so we surmised that PAR-1 and PAR-2 activation in microglia may exert similar functions. Our results showed that PAR-1 exerted stage-specific beneficial and detrimental effects by suppressing PAR-2-stimulated secretion. PAR-1 stimulation decreased the release of BDNF during the early phase of the reactive response, suggesting that early PAR-1 receptor activation may inhibit neuroprotection. Conversely, later PAR-1 stimulation suppressed the release of inflammatory mediators, suggesting a beneficial anti-inflammatory effect. This may be partly explained by the fact that activation of PAR-1 induces the expression of suppressor of cytokine signaling-3 (SOCS-3) in microglia [33]. Upon injury, up regulated cytokines act as proinflammatory agents as a part of a defense mechanism, but excessive or prolonged activation could be detrimental to the CNS. SOCS-3 is a crucial mediator which executes tight control of the strength and duration of these cytokine-triggered signaling pathways. Previous studies showed that deletion of SOCS3 in adult retinal ganglion cells promotes robust regeneration of injured optic nerve axons and up regulates the release of ciliary neurotrophic factor [34,35]. So, we postulated that during early phase of injury, transient up regulation of SOCS3, which aims to protect against further tissue damage by limiting production of inflammatory factors might inhibit 2-Furoyl-LIGRLO-NH2-induced BDNF release in microglial cells. However, SOCS3 has a short half-life (1 to 2 h) and its stability can be regulated by other proteins [36]. For these, at the later stage, the anti-inflammatory effects of SOCS3 are limited. Thus, at the later stage, activation of SOCS3 would exert its beneficial effects by limiting inflammation and suppressing immune responses. Futher, PAR1 which induces the expression of SOCS3 in microglia, has been proved to switch from being a detrimental to a benefical receptor during the progression of sepsis in mice [20]. The functional interactions between PAR-1 and PAR-2 in microglia underscore the ubiquity of these receptor complexes or co-factoring that occurs with many members of the PAR family.

In conclusion, the present study revealed that activation of microglia by PAR-2 stimulation triggered the sequential release of neuroprotective and neurotoxic factors, with early transient release of BDNF and delayed sustained release of NO, IL-1β, and TNF-α. Increased release of NO, IL-1β, and TNF-α at the late phase of PAR-2 stimulation could mediate apoptotic cell death of the neuron. Stage-specific blockade or activation of PAR-1 altered the amounts of neurotrophins and cytokines released in response to PAR-2 stimulation. PAR-1 antagonism increased the release of BDNF at the early phase (by 11%, small but significant), while PAR-1 agonism reduced the release of proinflammatory mediators during the late phase. These distinct temporal profiles could be exploited to develop novel therapeutic strategies for CNS injury.

## Abbreviations

ANOVA, analysis of variance; BDNF, brain-derived neurotrophic factor; CNS, central nervous system; DAPI, 4,6-diamidino-2-phenylindole; ELISA, enzyme-linked immunosorbent assay; FACS, fluorescence activated cell sorting; IL-1β, interleukin 1 beta; JNK, ERK1/2, c-Jun N-terminal kinase; MAPK, mitogen-activated protein kinase; MCM, microglia conditioned medium; PI, propidium iodide; qRT-PCR, quantitative real-time polymerase chain reaction; ROS, reactive oxygen species; SD, Sprague Dawley; SE, standard error; SOCS-3, suppressor of cytokine signaling-3TNF-α, tumor necrosis factor alpha; TUNEL, transferase-mediated dUTP nick-end labeling.

## Competing interests

The authors have no conflicts or competing interests.

## Authors’ contributions

The work presented here was carried out in collaboration between all authors. HY and DS defined the research theme. CC, QC and QO carried out most of the laboratory experiments, analyzed the data, interpreted the results and wrote the paper. JS designed methods and experiments. FL discussed analyses. All authors have read and approved the final manuscript.
